# Chemotherapy of Capecitabine plus Temozolomide for Refractory Pituitary Adenoma after Tumor Resection and Its Impact on Serum Prolactin, IGF-1, and Growth Hormone

**DOI:** 10.1155/2022/8361775

**Published:** 2022-03-21

**Authors:** Xirui Wang, Changwei Hu, Yabin Li, Baowen Ren, Gangfeng Yin

**Affiliations:** Third Department of Neurosurgery, Cangzhou Central Hospital, Cangzhou, Hebei Province, China

## Abstract

**Objective:**

To investigate the efficiency of capecitabine (CAP) plus temozolomide (TEM) in refractory pituitary adenoma after tumor resection and its impact on serum prolactin (PRL), insulin-like growth factor 1 (IGF-1), and growth hormone (GH) levels.

**Methods:**

From January 2017 to January 2020, 80 patients assessed for eligibility receiving transsphenoidal tumor resection for refractory pituitary adenoma in the Department of Neurosurgery of our hospital were recruited. They were randomly distributed at a ratio of 1 : 1 via the random number table method to receive either bromocriptine and TEM (control group) or bromocriptine plus combination chemotherapy of TEM and CAP (study group). The two groups were compared in terms of clinical efficacy and serum levels of PRL, IGF-1, and GH.

**Results:**

The objective response rate (ORR) was 87.50% and 67.50% in the study group and the control group, respectively (*P*=0.032). Before treatment, two groups had similar levels of PRL, IGF-1, and GH. After treatment, PRL levels in the study group were lower than that in the control group (278.35 ± 39.25 versus 326.35 ± 42.45, *P* < 0.001). Compared with the control group, IGF-1 levels in the study group were also lower (311.78 ± 28.82 versus 364.35 ± 31.35, *P* < 0.001). The study group presented markedly lower levels of thyroid-stimulating hormone (TSH) and higher serum levels of free thyroxine-4 (FT-4) and adrenocorticotropic hormone (ACTH) versus the control group (*P* < 0.05). The incidence of adverse events was comparable between the study group (30.0%) and the control group (22.5%) (*P* > 0.05). All eligible patients had similar progression-free survival (PFS) after chemotherapy.

**Conclusion:**

For patients with refractory pituitary adenoma, the combination chemotherapy of CAP and TEM significantly improves clinical outcomes and corrects hormonal disturbances, with a good safety profile, but its long-term efficacy requires further investigation.

## 1. Introduction

Pituitary adenoma (PA) is the most common intracranial tumor, in which most PAs grow benignly and are curable with surgery or medication [1]. However, a small proportion of PA shows rapid growth, being resistant to surgery, drugs, and radiotherapy, and recurs or regrows despite surgical resection with curative intent, known as a refractory pituitary adenoma (rPA) [2]. Research has shown an increase in the population prevalence of PA from 7.5 to 15/100,000 to 77.6/100,000, of which about 35% are rPA [3]. It seriously compromises patients' health and even endangers their lives, so its treatment remains a pressing issue in the clinical management of neurosurgery [4]. Transsphenoidal tumor resection is the preferred surgical approach for PA, and the combination with various techniques such as microscopy, neuroendoscopy, and neuronavigation shows significant enrichments in the safety and efficiency of the surgery [5]. Nonetheless, in addition to tumor resection, rPA also requires postoperative adjuvant medications to promote normal hormone secretion and ameliorate clinical outcomes [6].

Temozolomide (TEM) is an oral alkylating agent class whose active product transfers methyl to the O6 and N7 positions of DNA guanine, impeding DNA replication, arresting the cell cycle, and exerting antitumor functions [7]. The 2018 edition of the European Society of Endocrinology Guidelines recommends TEM as a first-line chemotherapeutic agent for refractory pituitary tumors and pituitary cancer [8]. Capecitabine (CAP) is a class of oral anticancer drugs that are rapidly absorbed via the intestinal mucosa after oral administration, converted to inactive intermediate 5′-deoxy-5′fluorocytidine by carboxylesterase in the liver, transformed to 5′-deoxy-5′fluorouracil by the action of cytidine deaminase in the liver and tumor tissue, and finally catalyzed by thymidine phosphorylase in the tumor tissue to fluorouracil (5-FU) to perform antitumor effects [9]. It is mainly used in advanced breast and colorectal cancers and also serves as salvage therapy for breast cancer after failure of anthracycline and paclitaxel therapies [10]. It has been suggested that the CAPTEM regimen showed significant benefits in the treatment of neuroendocrine tumors (NETs), but its application in rPA has been marginally explored [11]. Accordingly, the present study was to evaluate the efficacy of CAPTEM after transsphenoidal tumor resection for rPA.

## 2. Materials and Methods

### 2.1. Baseline Data

From January 2017 to January 2020, 80 patients assessed for eligibility receiving transsphenoidal tumor resection for refractory pituitary adenoma in the Department of Neurosurgery of our hospital were recruited. They were randomly distributed at a ratio of 1 : 1 via the random number table method to the control group (*n* = 40) or the study group (*n* = 40). The baseline data were collected via an interview with patients, including age, gender, and duration of disease. All patients provided written informed consent, and the study was conducted as per the Declaration of Helsinki principles, 2013 [12]. The protocol was ratified by our hospital' ethics committee.

### 2.2. Inclusion and Exclusion Criteria

#### 2.2.1. Inclusion Criteria

The inclusion criteria were as follows: aged 18–80 years old, diagnosed with PA by imaging and pathological examination, after transsphenoidal tumor resection, invasive growth of tumors on postoperative reexamination imaging, and reoperation is difficult for total resection, and Karnofsky performance scores (KPS) of 60 points or more.

#### 2.2.2. Exclusion Criteria

The exclusion criteria were as follows: with distant or intraspinal metastases on imaging, with comorbidities such as severe pulmonary hypertension, cardiovascular disease, peripheral vascular disease, and severe chronic heart disease that may affect the conduct of radiotherapy, with other malignancies, pregnant or fertile women, active mental disorders or other disorders that compromise the patient's cognitive function.

### 2.3. Treatment Methods

#### 2.3.1. Control Group

The control group was given oral administration of bromocriptine (manufacturer: Novartis Pharmaceutical Factory, approval number: State Drug Quantifier H20020370, specification: 1.25 mg/tablet) in combination with TEM (manufacturer: Jiangsu Tianshi Li Diyi Pharmaceutical Co., Ltd., approval number: State Drug Administration H20040637, specification: 50 mg/capsule). Bromocriptine: the starting oral dose was 1.25 mg/d and was increased gradually to a daily maintenance dose of 2 tablets/d, 3 times/d, with the maximum daily dose no more than 15 mg, for a total of 3 months of treatment. TEM was administered orally on an empty stomach with a starting dose of 150 mg/dose, once/day for 5 d, followed by an interval of 23 d, with 28 d as a cycle. If no documented adverse events were recorded in the first cycle, the dose was increased to 200 mg from the second cycle, for a total of 3 cycles of treatment.

#### 2.3.2. Study Group

On top of the treatment given to the control group, the study group was additionally given CAP (manufacturer: Shanghai Roche Pharmaceutical Co., Ltd., approval number: State Drug Administration H20073024, specification: 500 mg/capsule). CAP was taken within 30 minutes after meal with water. The dose was 1250 mg/dose, once in the morning and once at night daily, with an interval of 1 week after 2 weeks of treatment, 3 weeks as one course of treatment for a total of 4 courses.

### 2.4. Outcome

#### 2.4.1. Clinical Efficacy

The change of tumor volume under MRI/CT before and after treatment was recorded. According to tumor volume, clinical symptoms, and hormone levels, the clinical efficacy was stratified into complete response (CR), partial response (PR), stable disease (SD), and progressive disease (PD). CR: tumor and clinical symptoms disappear, and hormone levels return to the normal range. PR: tumor volume reduces by >20%, hormone levels decrease by >20%, and clinical symptoms show remission. PD: tumor volume increases, high hormone levels continue to rise, and clinical symptoms progress. SD: between PR and PD. Objective response rate (ORR) = (CR + PR)/total cases.

#### 2.4.2. Hematological Indexes

Five mL of fasting venous blood was collected from both groups before and after treatment and centrifuged at 3500 r/min for 10 min to collect the serum. Serum pituitary prolactin (PRL) was determined using electrochemiluminescence, serum insulin-like growth factor 1 (IGF-1) was determined using enzyme-linked immunosorbent assay (ELISA), serum growth hormone (GH) was determined using radioimmunoassay, and serum thyroid-stimulating hormone (TSH), free thyroxine-4 (FT-4), and adrenocorticotropic hormone (ACTH) were determined by electrochemiluminescence.

#### 2.4.3. Adverse Events

Adverse effects such as gastrointestinal reactions, ototoxic reactions, and neurotoxic reactions were recorded in both groups during the treatment to calculate the incidence of adverse events.

#### 2.4.4. Long-Term Efficacy

Postoperative follow-up was performed once a month for a total of 12 months, and the progression-free survival (PFS) of patients in both groups was recorded to plot the PFS curves.

### 2.5. Statistical Analysis

SPSS 23.0 software was used for data analyses, and GraphPad Prism 9.0 software was used to plot the images. The measurement data were expressed as mean ± standard deviation (±*s*), and differences between groups were examined by the *t*-test. The count data were expressed as rate (*n*%) and the chi-square test was used to verify the presence or absence of statistical differences. The Kaplan–Meier curve was used to compare the difference of progression-free rate between two groups. Differences were considered statistically significant at *P* < 0.05.

## 3. Results

### 3.1. Baseline Data

The two groups presented comparable baseline data such as gender, age, course of disease, and pathological types (*P* > 0.05) (Table 1).

### 3.2. Clinical Efficacy

The control group had 12 cases of CR, 15 cases of PR, 9 cases of SD, and 4 cases of PD, with an ORR of 67.5% (27/40). The study group had 16 cases of CR, 19 cases of PR, 3 cases of SD, and 2 cases of PD, with an ORR of 87.50% (35/40). Combination chemotherapy of CAPTEM achieved significant improvements in clinical efficacy versus mono chemotherapy of TEM (*P*=0.032) (Table 2).

### 3.3. Hormone-Related Indexes

Before treatment, the two groups had similar levels of PRL, IGF-1, and GH. After treatment, the above indices showed a significant decline, with lower results observed in the study group (*P* < 0.001) (Table 3).

### 3.4. The Levels of TSH, FT-4, and ACTH

Before treatment, the two groups showed no significant differences in the levels of TSH, FT-4, and ACTH (*P* > 0.05). The study group presented markedly lower levels of TSH and higher serum levels of FT-4 and ACTH versus the control group (*P* < 0.05) (Table 4).

### 3.5. Adverse Events

The control group had 4 cases of gastrointestinal reactions, 1 case of ototoxicity, 2 cases of neurotoxicity, and 2 cases of other adverse events, with an overall incidence of 22.50% (9/40). The study group had 3 cases of gastrointestinal reactions, 2 cases of ototoxicity, 3 cases of neurotoxicity, and 4 cases of other adverse events, with an overall incidence of 30.00% (12/40). No significant differences in adverse events were observed between the two groups (*P*=0.446) (Table 5).

### 3.6. Long-Term Efficacy

**Figure 1:**
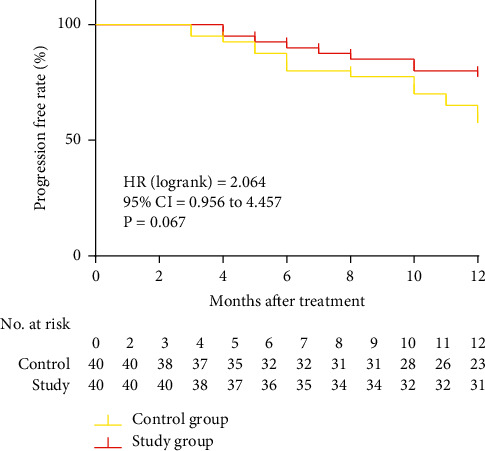
PFS curve by Kaplan–Meier.

No loss to the 12-month follow-up was recorded in all eligible patients. The 6-month and 12-month PFS of patients in the control group were 80.00% and 67.5%, respectively, and those of the study group were 87.50% and 77.5%, respectively (*P* > 0.05) (Table 6). There was no significant difference in the 12-month Kaplan–Meier PFS analysis between the two groups of patients (*P*=0.067) (Figure 1).

## 4. Discussion

Refractory pituitary adenoma (rPA) is a special type of pituitary tumor, with undefined incidence, diagnostic criteria, and proper treatment methods, which poses a serious challenge for diagnosis and treatment [12, 13]. Surgical excision of tumor tissue can reduce the compression of intracranial tissues such as the optic nerve and hypothalamus, relieve the symptoms of hydrocephalus and severe headache, correct endocrine dysfunction, and preserve normal pituitary function. Most rPAs exhibit aggressive growth and invade adjacent structures such as bone, dura mater, and brain tissue, compounding the difficulty of complete resection [14]. Here, bromocriptine combined with TEM was used as a basic treatment for rPA after tumor resection. Bromocriptine, a derivative of ergometrine, is a specific allosteric hypothalamic and pituitary dopamine receptor agonist that acts directly on the adenohypophysis to inhibit PRL secretion and facilitate GH release [15]. It has been reported that bromocriptine is effective in the treatment of hyperprolactinemia with prolactinoma, as bromocriptine effectively regulates prolactin, reestablishes gonadal function, and reduces tumor volume [16]. TEM is a second-generation alkylating antitumor agent, a cell cycle nonspecific agent that inhibits tumor cell growth at all stages and is suitable for slow-growing tumors such as PA, which is therefore considered the first-line chemotherapeutic agent for rPA. The overall efficiency of oral TEM 150–200 mg/d in rPA patients reached approximately 47% (95% CI: 36%–58%) in a previous study [17]. TEM can play a cytotoxic role by alkylating the oxygen atom at position 6 and the nitrogen atom at position 7 of guanine on the DNA molecule, thereby eliciting apoptosis in tumor cells through mismatch repair of methylation adducts [18]. It has been demonstrated in various studies as the drug of choice for salvage treatment of rPA [19]. The combination of TEM and bromocriptine herein also achieved good results in significantly decreasing the postoperative levels of PRL, IGF-1, and GH and enhancing the pituitary function.

In the present study, CAPTEM showed significant benefits in clinical efficiency and the enhancement of hormone levels and pituitary function. CAP is an alternative drug to 5-F uracil (5-FU) that acts in tumor tissue catalyzed by thymidine phosphorylase to 5-FU [20]. It has been reported that CAPTEM demonstrated significant activity in the treatment of neuroendocrine tumors (NETs), particularly in pancreatic NETs, with ORRs ranging from 30% to 70% and improved PFS and overall survival (OS) of patients [21, 22]. In vitro data from NETs cell lineage suggest a synergistic effect of the CAPTEM regimen, but its use in refractory pituitary tumors has only been reported on a case-by-case basis, with no evidence supporting its advantages [23]. The pituitary gland is an important endocrine organ, and the development of PA is accompanied by endocrine changes, and serum PRL, IGF-1, and GH determinations are the main basis for the diagnosis of pituitary tumors [24]. Changes in serum TSH, FT-4, and ACTH, which are indicators of pituitary function, can reflect pituitary function [25]. The results of this study confirmed that CAPTEM regimen significantly enhanced ORR, corrected endocrine disorders, and improved pituitary function in patients, which provide a basis for its application in clinical practice. Furthermore, the absence of differences between the two groups indicates a good safety profile of the CAPTEM regimen. However, the 12-month follow-up results showed no improvements in patients' survival, which may be attributable to the small sample size and the short follow-up duration.

To sum up, for patients with refractory pituitary adenoma, the combination chemotherapy of CAP and TEM significantly improves clinical outcomes, corrects hormonal disturbances, and enhances the pituitary function, with a good safety profile, but its long-term efficacy requires further investigation.

## Figures and Tables

**Table 1:** Comparison of the baseline data.

	Control group (*n* = 40)	Study group (*n* = 40)	*t*/*χ*^2^	*P*
Age ($\bar{x}\pm s$, years)	54.55 ± 12.36	56.18 ± 14.02	0.550	0.584
Gender (*n*)			0.205	0.651
Male	16	18		
Female	24	22		
Course of disease ($\bar{x}\pm s$, months)	16.25 ± 4.46	15.03 ± 3.26	1.401	0.165
CTV of resection	1.78 ± 0.42	1.81 ± 0.54	0.275	0.784
KPS scores	77.31 ± 10.82	79.11 ± 11.45	0.721	0.473
Pathological types			0.572	0.751
Prolactinoma	14	11		
Acromegaly	16	17		
Cushing's disease	10	12		

CTV, clinical tumor volume.

**Table 2:** Clinical efficacy.

	CR	PR	SD	PD	ORR
Control group (*n* = 40)	12	15	9	4	27 (67.50)
Study group (*n* = 40)	16	19	3	2	35 (87.50)
*χ* ^2^					4.588
*P*					0.032

CR, complete response; PR, partial response; SD, stable disease; PD, progressive disease; ORR, overall response rate.

**Table 3:** Serum levels of PRL, IGH-1, and GH ($\bar{x}\pm s$, ng/mL).

	PRL		IGF-1		GH	
	Before	After	Before	After	Before	After
Control group (*n* = 40)	372.44 ± 34.21	326.35 ± 42.45	468.38 ± 46.26	364.35 ± 31.35	13.54 ± 3.13	8.34 ± 2.14
Study group (*n* = 40)	384.15 ± 40.32	278.35 ± 39.25	455.25 ± 52.17	311.78 ± 28.82	14.03 ± 4.21	5.44 ± 1.46
*t*	1.400	5.251	1.191	7.808	0.581	7.078
*P*	0.165	<0.001	0.237	<0.001	0.563	<0.001

PRL, prolactin; IGF-1, insulin-like growth factor 1; GH, growth hormone.

**Table 4:** Serum levels of TSH, FT-4, and ACTH $\left(\bar{x}\pm s\right)$.

	TSH		FT-4		ACTH	
	Before	After	Before	After	Before	After
Control group (*n* = 40)	9.35 ± 2.35	4.62 ± 1.46	8.93 ± 2.03	13.35 ± 3.11	1.56 ± 0.46	7.34 ± 2.15
Study group (*n* = 40)	10.14 ± 3.02	3.27 ± 1.24	9.15 ± 2.16	14.02 ± 3.62	1.63 ± 0.51	9.03 ± 3.11
*t*	1.307	4.436	0.485	0.887	0.668	2.827
*P*	0.195	≤0.001	0.629	0.378	0.506	0.006

**Table 5:** Adverse events during treatment.

	Gastrointestinal reactions	Ototoxicity	Neurotoxicity	Others	AEs
Control group (*n* = 40)	4	1	2	2	9 (22.50)
Study group (*n* = 40)	3	2	3	4	12 (30.00)
*χ* ^2^					0.581
*P*					0.446

AEs, adverse events.

**Table 6:** 6-month and 12-month PFS.

	6 months	12 months
Control group (*n* = 40)	32 (80.00)	27 (67.5%)
Study group (*n* = 40)	35 (87.5%)	31 (77.5%)
*χ* ^2^	0.827	1.003
*P*	0.363	0.317

## Data Availability

The datasets used during the present study are available from the corresponding author upon request.
